# Cerebellar Stroke in a Young Adult Following an Amusement Park Ride

**DOI:** 10.7759/cureus.44165

**Published:** 2023-08-26

**Authors:** Syed Hashim Ali Inam, Muhammad Tayyab Muzaffar Chaychi, Muhammad Shaheer Mannan, Jonathan E Hanson, Paul Ferguson, Justin Nolte

**Affiliations:** 1 Neurology, Joan C. Edwards School of Medicine, Marshall University, Huntington, USA; 2 Neurology, Quaid-e-Azam Medical College, Bahawalpur, PAK; 3 Neurology, CMH Lahore Medical College and Institute of Dentistry, Lahore, PAK

**Keywords:** stroke of unknown etiology, atypical stroke, stroke in young, stroke, amusement park ride, cerebellar stroke

## Abstract

The incidence of stroke in young individuals has been showing an increasing trend. In such cases, atypical mechanisms of stroke should be considered. Here, we report a case of a 37-year-old healthy female who presented with complaints of right-sided ataxia and clumsiness that started after an amusement park ride where she was swung rapidly. Imaging revealed an infarct in the right cerebellar hemisphere. This case report highlights certain mechanisms that can cause a stroke in this situation, with special emphasis on recognition, timely medical management, public awareness of such risk factors, and the prevention of complications.

## Introduction

The global lifetime risk of stroke increased by 8.9% from 1990 to 2016 [1]. In the United States, stroke is the fifth leading cause of death, and the overall stroke prevalence is estimated to be around 2.5% [1,2]. Stroke mortality data in young and middle-aged adults shows an increasing trend despite a decline in the age-standardized stroke incidence rate from 1990 to 2016 in the overall population [3-5]. This is further supported by increasing rates of hospitalizations for young patients with acute ischemic stroke [6,7]. 

Modifiable risk factors (high blood pressure, obesity, hyperglycemia, and hyperlipidemia) may be attributed to around 90% of stroke risk, while behavioral risk factors (10-pack-year history of smoking, sedentary lifestyle, and an unhealthy diet) account for 74% of the risk [2]. Stroke is traditionally considered a disease of the middle-aged and elderly, and as a result, these age groups are often the most studied, and data regarding stroke in young individuals under 45 years of age is limited. Moreover, given that the prevalence of cardiovascular risk factors has been stable in recent years, wide-scale and long-term data specifically tailored to risk factors in young individuals is needed to better understand the unique risk factors for stroke in these patients [3,4]. This is further supported as 'stroke of undetermined etiology' remains the leading cause of stroke in young adults classified according to the trial of ORG 10172 in acute stroke treatment (TOAST) criteria [8,9]. In young patients without typical risk factors for stroke, many pathophysiological mechanisms have been hypothesized as an underlying cause. For example, cervical artery dissection has been reported to contribute to 10% to 25% of all strokes in young adults [10]. Similarly, up to 45% of young patients with cryptogenic stroke have a patent foramen ovale, and multiple meta-analyses have shown decreased recurrence of stroke in patients undergoing foramen ovale closure [11,12].

Several cases of patients who experience acute ischemic stroke after rollercoaster or amusement park rides have been described in the literature with varying etiologies, including carotid compression, immature cervical spine development, and vertebral artery dissection [13-15]. However, fewer than 20 cases of cerebrovascular accidents following such acceleration-based amusement park rides have been reported. Along with a literature review of ischemic stroke after amusement park rides, we present the case of a young female with a history of smoking and hypertension who presented to the emergency department with a headache, right-sided clumsiness, and gait ataxia that started after a ride in an amusement park and was eventually diagnosed with a cerebellar ischemic stroke.

## Case presentation

A 37-year-old female with a past medical history of untreated hypertension (on lisinopril, non-compliant) and a long-standing history of tobacco use (one pack of cigarettes a day for 10 years) presented to the ED with complaints of right-sided clumsiness and gait ataxia that started after an amusement park ride in a local county fair, in which she had been swung in a circular direction. Symptom onset was on a Friday afternoon, as she was in the middle of an amusement park ride. A head injury was not reported before or after the ride. She was immediately driven to the ED by her husband. The external ED diagnosed her with vertigo and discharged home with anti-emetics. However, by Sunday, two days after the initial event, her symptoms had not improved, and her home blood pressure remained significantly elevated. Therefore, her husband brought her to our ED for evaluation.

**Figure 1:**
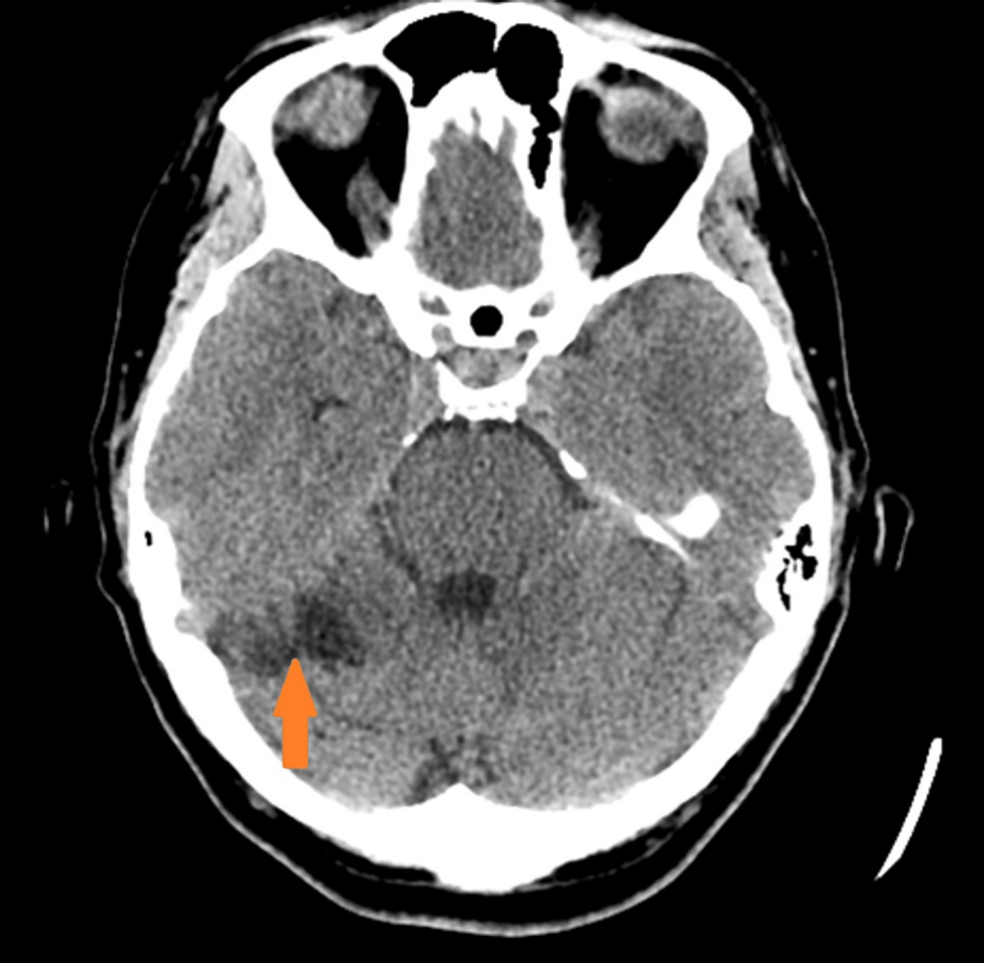
CT head on day 3 showing the right superior cerebellar artery infarct

While in our ED, her blood pressure was 199/82, heart rate was 82, respiratory rate was 18, and oxygen saturation was 97%. Her basic labs were unremarkable. A non-contrast CT head was ordered and reported as an infarct in the right cerebellar hemisphere, superior cerebellar artery territory (Figure 1).

A subsequent CT angiogram of the head and neck reported a right cerebellar infarct in the right superior cerebellar artery region. The proximal right superior cerebellar artery was patent; however, the distal aspect was not well visualized. Proximal vertebral arteries were without significant stenosis or abnormality to suggest a source of her apparent embolic stroke.

Neurology was consulted; on physical examination, she had right-sided ataxia and right-sided dysmetria in her upper and lower extremities. Power was 5/5, reflexes were 2+, and no sensory abnormalities were found on the exam. The patient was given aspirin 325 mg stat and started on aspirin 81 mg daily. She was also started on atorvastatin 40 mg at bedtime. 

She was admitted to the neurology service for further evaluation. Stroke labs revealed A1c 5.2%, B12 479 pg/mL, low-density lipoprotein (LDL) 109 mg/dL, thyroid-stimulating hormone (TSH) 1.020 mU/L, and negative for beta-2 glycoprotein and cardiolipin antibodies. Factor V protein and methylenetetrahydrofolate reductase (MTHFR) testing were normal. The hypercoagulable profile was positive for cardiolipin IgM but negative for cardiolipin IgG. Polycystic ovaries were ruled out. The patient complained about a post-stroke headache that was controlled with acetaminophen. She was discharged home with outpatient physical therapy follow-up. 

Her symptoms improved at her outpatient follow-up visit, but the headache persisted. She was started on rimegepant, and her headache resolved. The patient reported no new stroke symptoms and stated that she had quit smoking. The patient denied a history of stroke, cardiovascular disease, sickle cell disease, or hypercoagulable disorders in her family.

**Figure 2:**
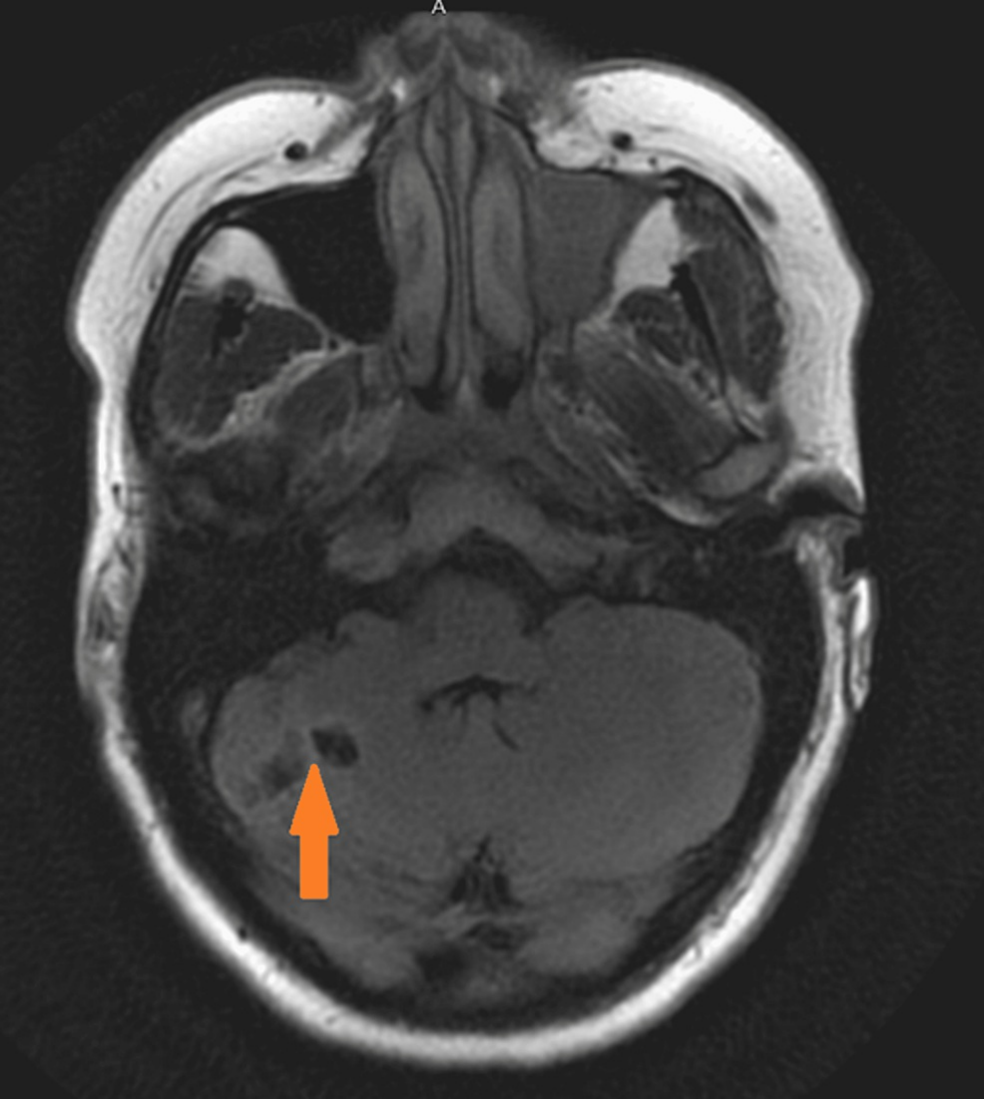
MRI of the brain one month after the incident showing the right cerebellar infarct

The patient had worsening ataxia after her discharge; therefore, a repeat MRI of the brain without contrast was ordered to evaluate for stroke burden and to look for a new stroke after one month of the event. This revealed an old infarct in the right cerebellar hemisphere and showed no other areas of embolic stroke outside the right subclavian artery (RSCA) territory of ischemic stroke (Figure 2).

**Figure 3:**
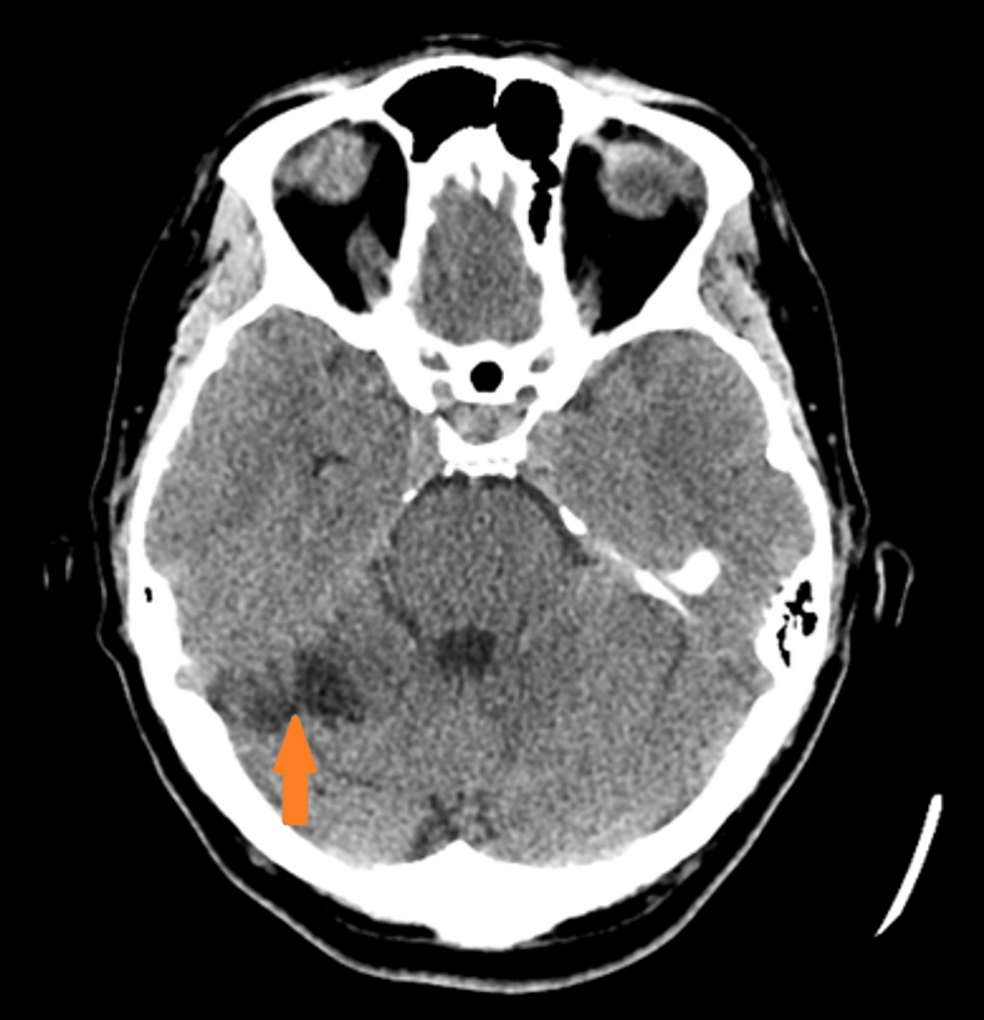
CT head showing the right cerebellar infarct three months after the incident

A CT angiogram (CTA) of the head and neck performed six months later revealed an old right cerebellar infarct and an unruptured right middle cerebral artery (MCA) bifurcation aneurysm measuring up to 4 mm that was stable (Figure 3). A six-month follow-up CTA head and neck was recommended by radiology for monitoring of the right MCA bifurcation aneurysm that was stable.

## Discussion

Studies to quantify forces generated during rotational acceleration of the head and their potential for traumatic brain injuries have been limited by the number of participants, the use of mathematical modeling only, or not being able to study the potential for injury other than mechanical deformations [16,17]. Moreover, head motion and brain deformation during rollercoaster rides have been shown to vary considerably between individuals [18]. Furthermore, with improving material science and technology, the average force experienced during amusement rides has increased considerably over the last 40 years [19]. In our case, the patient had been on a newer ride at a local county fair that swung her rapidly in a circular direction. 

Amusement parks usually display warnings for certain individuals and people at both extremes of age. These include people with cardiac conditions, high blood pressure, pregnant women, and those who suffer from motion sickness or dizziness. However, warnings about possible neurological sequelae of such rides are uncommon. Therefore, neurological symptoms often go unrecognized, leading to a delay in seeking medical treatment [13]. Hence, prompt recognition of these symptoms via public education about the common signs of stroke via the widely accepted mnemonic regarding symptoms of stroke, i.e., BEFAST, and advice to immediately seek care can lead to better outcomes in these patients. Similarly, providers should be cognizant of the possibility of various types of neurological injuries associated with these amusement park rides, which include subdural hematoma, subarachnoid hemorrhage, intraparenchymal hemorrhage, cervicocephalic arterial dissection, carotid artery thrombosis with stroke, and posttraumatic migraine [20].

Neck hyperextension is a frequent consequence of rollercoaster rides. Studies have demonstrated that the internal carotid artery experiences peak stress levels at 90 degrees of lateral rotation or 45 degrees of hyperextension [21]. Vertebral artery dissection (VAD) can arise due to rotational forces affecting the artery's third segment at the C2 and C1 vertebral levels. This dissection causes stenosis of the true lumen and disruption of the artery's epithelium. Subsequently, slow flow, turbulence, or irregularity in the endothelium can trigger the formation of local blood clots, potentially leading to artery-to-artery embolism, commonly affecting the posterior cerebral artery [22]. Furthermore, dissection has also been described following a minor injury such as chiropractic manipulation or a fall from a skateboard and occasionally occurs spontaneously or without trauma as well [23]. The standard neuroimaging for the detection of cervical arterial intramural hematoma includes magnetic resonance angiography (MRA) with fat-suppressed T1 axial images (without contrast) through the neck. This imaging modality reveals a characteristic crescent sign of hyperintensity in the wall of the dissected vessel. The gold standard for visualizing vertebral artery dissections is conventional cerebral angiography. Conventional cerebral angiography may demonstrate focal dilation, proximal or distal stenosis, or aneurysmal dilatation in patients with vertebral artery dissections [15]. While the MRA protocol and conventional angiogram are the gold standard modalities for confirmation of dissections, we opted against further diagnostic testing in our patient after reviewing the CT angiogram with neuroradiology and their feeling that subsequent testing was not necessary to evaluate the dissection in her case.

While the likelihood of diffuse white matter axonal injury is low due to the lower forces generated by amusement rides compared to motor vehicle accidents and other forms of high-G force activities, the potential for other vascular injuries remains high, as evidenced by reported cases in the literature. Our search (Table 1) uncovered 15 instances of acute ischemic stroke or dissection leading to ischemia resulting from amusement park or rollercoaster rides. Among these cases, eight were male and seven were female. The pediatric population accounted for seven cases, while adults less than 45 years of age made up the remaining eight. The stroke incidents were distributed as follows: six cases involved the anterior circulation, eight involved the posterior circulation, and one affected both the anterior and posterior circulations. Notably, vertebral artery dissection emerged as the most common underlying cause of stroke in these patients. Therefore, vertebral artery dissection should always be considered a significant differential when a young or old patient presents with ischemic symptoms without the typical risk factors for stroke. These symptoms could range from headache, vertigo, diplopia, visual changes, ataxia, vomiting, sensory changes, and vomiting that correlates with an ischemic infarct in one of the vascular distributions. Vascular injury following acceleration or deceleration situations should be a priority, as recognizing vascular dissections of an internal carotid artery is time-sensitive due to the high potential for the development of a subsequent hemispheric stroke [13]. Furthermore, clinicians actively involved in the care of such patients should be aware of the wide array of clinical presentations and vascular territories that may be involved, thus exercising a high degree of suspicion. 

**Table 1:** Details of case reports of ischemic stroke and/or arterial dissections following rides in amusement parks or rollercoasters

First author	Year	Sex	Age (in years)	Clinical symptoms	Diagnosis
Scheer [15]	1979	Female	13	Headache, right-sided hemiparesis, and difficulty speaking	Left internal carotid artery occlusion
Biousse [24]	1995	Female	31	Vertigo, headache, and neck pain	Left vertebral artery dissection
Burneo [22]	2000	Male	30	Diplopia, cervical pain	left occipital infarction and left vertebral artery dissection
Lascelles [25]	2001	Male	11	Sudden headache, hemiparesis, ataxia, dizziness, nausea, and vomiting	Left cerebellar infarct, infarction of thalami, and vertebral artery dissection
Blacker [26]	2003	female	41	Right frontotemporal headache and drooping of the right eye	Right internal carotid artery dissection
Schneck [13]	2008	Female	34	Neck pain, vertigo, blurred vision, and middle horizontal diplopia	Bilateral cervical and vertebral dissections
Arat [27]	2011	Female	35	Right-sided neck pain and frontal headache	Bilateral Internal carotid artery and vertebral artery dissections
Leitao [28]	2012	Male	22	Occipital headache, vertigo, nausea, vomiting and ataxia	Infarct of the right cerebellum and dissection of the right vertebral artery at C1–C2
Kurita [29]	2014	Male	39	Headache, sudden right-sided hemiplegia, and aphasia	Infarction of left middle cerebral artery territory, Internal carotid artery, middle cerebral artery dissection
Nouh [21]	2015	Male	4	Left facial droop, vomiting, inability to walk, and left-sided weakness	Right middle cerebral artery infarction and right internal carotid artery dissection
Sorrentino [30]	2015	Male	8	Headache, blurred vision, and bilateral superior quadrantanopia	Bilateral occipital infarction
Baumgartle [14]	2016	Male	12	Headache, facial droop, slurred speech, and right-sided weakness	Left middle cerebral artery infarction
Whitcomb [31]	2017	Female	13	Confusion, disorientation, right-sided facial droop, and right-sided weakness	Dissection of the left internal carotid artery
Alboudi [32]	2018	Male	16	Headache, vertigo, and neck pain	Left vertebral artery dissection with occlusion of the left posterior inferior cerebellar artery
Gomez-Rojas [33]	2020	Female	37	Headache and neck pain	Bilateral vertebral artery dissection

## Conclusions

In conclusion, this case report and literature review underscore the importance of recognizing acute ischemic stroke as a potential consequence of amusement park rides in young adults and children. Prompt recognition of stroke symptoms, timely medical intervention, and public awareness campaigns are essential for better outcomes and the prevention of complications. Neck hyperextension and vertebral artery dissection emerge as prominent mechanisms in such stroke cases. Healthcare providers need to be vigilant in identifying and managing these cases, and further research on specific risk factors in young individuals is crucial to improving prevention and safety measures in amusement park settings.
